# Retraction: Structure based sequence analysis & epitope prediction of gp41 HIV1 envelope glycoprotein isolated in Pakistan

**DOI:** 10.1186/1479-0556-10-10

**Published:** 2012-10-23

**Authors:** Syyada Samra Jafri, Saliha Kiran, Syed Babar Jamal, Masaud Shah

**Affiliations:** 1 Bioinformatics Research Group, National Center of Excellence in Molecular Biology, University of the Punjab, Lahore, Pakistan; 2 Government College University, Faisalabad, Pakistan; 3 International Islamic University Islamabad, Islamabad, Pakistan

## 

This article [1] is retracted by the Editor because the peer review process was compromised due to a referee’s undeclared conflict of interest. Based on post-publication peer review the Editor cannot trust the veracity of the findings. We apologize to all affected parties.
